# Functional segregation and integration within fronto-parietal networks

**DOI:** 10.1016/j.neuroimage.2016.08.031

**Published:** 2017-02-01

**Authors:** Valeria Parlatini, Joaquim Radua, Flavio Dell’Acqua, Anoushka Leslie, Andy Simmons, Declan G. Murphy, Marco Catani, Michel Thiebaut de Schotten

**Affiliations:** a Sackler Institute of Translational Neurodevelopment, Department of Forensic and Neurodevelopmental Sciences, Institute of Psychiatry, Psychology and Neuroscience, King's College London, SE5 8AF London, UK; b Natbrainlab, Department of Forensic and Neurodevelopmental Sciences, Institute of Psychiatry, Psychology and Neuroscience, King's College London, SE5 8AF London, UK; c Department of Psychosis Studies, Institute of Psychiatry, Psychology and Neuroscience, King's College London, SE5 8AF London, UK; d FIDMAG Germanes Hospitalàries, CIBERSAM, Sant Boi de Llobregat 08035, Spain; e Department of Neuroimaging, Institute of Psychiatry, Psychology and Neuroscience, King's College London, SE5 8AF London, UK; f NIHR Biomedical Research Centre for Mental Health at South London and Maudsley NHS Foundation Trust and King's College London, Institute of Psychiatry, SE5 8AF London, UK; g MRC Centre for Neurodegeneration Research, King's College London, SE5 9RX London, UK; h Brain Connectivity Behaviour group, FrontLab, Paris, France; i Sorbonne Universités, UPMC Univ Paris 06, Inserm, CNRS, Institut du cerveau et la moelle (ICM) - Hôpital Pitié-Salpêtrière, Boulevard de l’hôpital, F-75013, Paris, France

**Keywords:** Frontal parietal, Functional magnetic resonance imaging (fMRI), Meta-analysis, Superior longitudinal fasciculus, Diffusion tractography

## Abstract

Experimental data on monkeys and functional studies in humans support the existence of a complex fronto-parietal system activating for cognitive and motor tasks, which may be anatomically supported by the superior longitudinal fasciculus (SLF). Advanced tractography methods have recently allowed the separation of the three branches of the SLF but are not suitable for their functional investigation. In order to gather comprehensive information about the functional organisation of these fronto-parietal connections, we used an innovative method, which combined tractography of the SLF in the largest dataset so far (129 participants) with 14 meta-analyses of functional magnetic resonance imaging (fMRI) studies. We found that frontal and parietal functions can be clustered into a dorsal spatial/motor network associated with the SLF I, and a ventral non-spatial/motor network associated with the SLF III. Further, all the investigated functions activated a middle network mostly associated with the SLF II. Our findings suggest that dorsal and ventral fronto-parietal networks are segregated but also share regions of activation, which may support flexible response properties or conscious processing. In sum, our novel combined approach provided novel findings on the functional organisation of fronto-parietal networks, and may be successfully applied to other brain connections.

## Highlights

• We used a novel approach to investigate fronto-parietal functions.
• These are segregated into a dorsal spatial and a ventral non-spatial network.
• These networks rely on the superior longitudinal fasciculus (1st and 3rd branch).
• They overlap on areas with flexible response properties that rely on the 2nd branch.

## 1 Introduction

Electrical recordings in monkeys revealed that fronto-parietal networks are essential for transforming sensory information into action. These networks work in parallel and are likely to specialise for different aspects of sensory-motor integration. For instance, previous authors suggested a subdivision into a medial network for preparation of action, a dorsolateral network for reaching, and a ventral network for grasping (Rizzolatti et al., 1998).

In humans, functional magnetic resonance imaging (fMRI) studies showed a similar dorso-ventral segregation of the frontal-parietal networks for several tasks, including voluntary and reflexive saccadic movements (Mort et al., 2003), spatial and verbal working memory (Rottschy et al., 2012), and voluntary oriented and stimulus-grabbed visuo-spatial attention (Corbetta and Shulman, 2002).

Preliminary studies suggest that this functional segregation may reflect the underlying anatomical separation of the fronto-parietal networks, which is mediated by the superior longitudinal fasciculus (SLF), a complex associative tract (Petrides and Pandya, 1984, Schmahmann and Pandya, 2006). However, the large majority of tractography studies considered the SLF as a single bundle, and often not clearly separated by the arcuate fasciculus, (Broser et al., 2012, Agosta et al., 2013, Myall et al., 2013, Abhinav et al., 2014, Kamali et al., 2014, Meyer et al., 2014), and thus limited possible investigations on its functional roles. Indeed, only recent advances in tractography, such as the spherical deconvolution algorithm we developed (Dell’acqua et al., 2010), enabled the visualisation of the entire anatomy of the SLF crossing through the corona radiata, thus allowing its subdivision into distinct components in the living human brain (Thiebaut de Schotten et al., 2011a).

Specifically, three different branches can be identified. A dorsal branch (SLF I) connects regions of the superior parietal lobule and superior frontal lobe. A middle branch (SLF II) connects regions of the intraparietal sulcus to regions of the superior and middle frontal gyrus. A ventral branch (SLF III) connects the inferior parietal lobule to the inferior frontal gyrus. This anatomical subdivision has been reported for both monkeys and humans (Petrides and Pandya, 1984, Makris et al., 2005, Thiebaut de Schotten et al., 2011a). Thus, spherical deconvolution provided an important advancement compared to previous approaches (Makris et al., 2005), but does not allow for the functional investigation of the identified branches.

In this study we tested the hypothesis that the revealed subdivision of the SLF in distinct anatomical components may underlie different functional roles, by combining tractography with a meta-analytic approach, which identified the fronto-parietal areas more likely to be involved in a specific function. This novel combined approach offered the advantage of exploring several functions at the same time, and of being independent from an *a priori* hypothesis regarding tract functions. Further, it allowed us not just to explore the anatomical and functional segregation of the fronto-parietal networks, which may hold true for some functions, but also the connectional anatomy underlying fronto-parietal regions subserving different tasks (Duncan, 2006, Dehaene and Changeux, 2011). In fact, it has been suggested that regions of overlap between dorsal and ventral networks belong to a core circuit that either adapts to represent the information of many tasks (the multiple demand pattern network (Duncan, 2006)) or mediates a modality-independent conscious access (Dehaene and Changeux, 2011).

In sum, in this study, we used advanced spherical deconvolution tractography to dissect the three branches of the SLF in the largest population of healthy controls so far; and combined tractography with 14 meta-analyses of fMRI studies as an innovative method to investigate the functional organisation of the identified white matter tracts.

## 2 Methods

### 2.1 Mapping of the SLF

We used diffusion tractography to identify the three branches of the SLF in 129 healthy right-handed volunteers (59 males and 70 females) aged between 18 and 79 years. For each participant, 60 contiguous near-axial slices were acquired on a 3 T GE Signa HDx TwinSpeed system (General Electric, Milwaukee, WI, USA) with the following parameters: rostro-caudal phase encoding, voxel size 2.4×2.4×2.4 mm, matrix 128×128, slices 60, NEX 1, TE 93.4 ms, b-value 3000 s/mm^2^, 60 diffusion-weighted directions and 7 non-diffusion-weighted volumes, using a spin-echo EPI sequence. Cardiac gating was applied with effective TR of 20/30 R-R intervals. Quality control of the data was assured using an automated analysis system (Simmons et al., 1999). Standard diffusion tensor tractography does not allow the reconstruction of the two most dorsal branches of the SLF because of the crossing of the dorsal association fibres with commissural and projection fibres (Thiebaut de Schotten et al., 2011a, Thiebaut de Schotten et al., 2011b, Rojkova et al., 2016).

Crossing problems can be partially overcome by more recent methods, such as diffusion spectrum imaging (DSI) (Wedeen et al., 2008) and high angular resolution diffusion imaging (HARDI) (Frank, 2001; Tournier et al., 2004; Dell’Acqua et al., 2007; Dell’acqua et al., 2010). For instance, the latter estimates a distribution of possible fibre orientations in the three-dimensional space for each voxel. The result is a function, whose multi-peak shape reflects the orientation and weight of each fibre component (Tournier et al., 2004; Anderson, 2005; Dell’Acqua et al., 2007; Dell’acqua et al., 2010). Among HARDI methods, tractography based on spherical deconvolution (SD) has been widely used to reconstruct white matter tracts in regions with multiple crossings, such as the SLF, which is object of the current investigation (Thiebaut de Schotten et al., 2011a, Thiebaut de Schotten et al., 2011b; Catani et al., 2012; Chechlacz et al., 2015; Marshall et al., 2015; Budisavljevic et al., 2016; Cazzoli and Chechlacz, 2016). A modified (damped) version of the Richardson-Lucy algorithm for spherical deconvolution (Dell’acqua et al., 2010) was employed using the software StarTrack (http://www.natbrainlab.co.uk). Algorithm parameters were chosen as previously described by our group (Dell’Acqua et al., 2013).

Whole brain tractography selected every brain voxel with at least one fibre orientation as a seed voxel. From these voxels, we reconstructed the streamlines by sequentially piecing together discrete and shortly spaced estimates of fibre orientation to form continuous trajectories (Dell’Acqua et al., 2013). When entering a region with crossing white matter bundles, the algorithm followed the orientation vector of least curvature (Schmahmann et al., 2007). Streamlines were halted when a voxel without fibre orientation was reached or when the curvature between two steps exceeded a threshold of 45°. The software estimating and reconstructing the orientation vectors and the trajectories from diffusion MRI was written in Matlab 7.8 (http://www.matwork.com).

Tractography dissections of the SLF I, II and III were performed using a multiple regions of interest (ROIs) approach: in each hemisphere three ROIs were delineated around the white matter of the superior, middle and inferior/precentral frontal gyri, and a ROI around the white matter of the parietal lobe. In order to exclude fibres belonging to the long and posterior segment of the arcuate fasciculus, which respectively connect frontal or parietal regions with temporal regions, a no-part ROI was delineated around the temporal white matter. Further details can be found in (Thiebaut de Schotten et al., 2011a).

For each participant, a convergence speed (CS) map of the deconvolution algorithm (Dell’acqua et al., 2006) was estimated. CS map quantifies how quickly the residual fitting error between the diffusion signal, and the fibre model as identified by the deconvolution algorithm decays within each voxel. CS maps better contrast white matter regions showing a smaller partial volume effect, as compared to FA or similar anisotropy maps. CS maps were registered to the MNI152 template provided with the FMRIB Software Library package (FSL, http://www.fmrib.ox.ac.uk/fsl/) using Advance Normalisation Tools (ANTs, http://www.picsl.upenn.edu/ANTS/), which combines affine with diffeomorphic deformations (Avants et al., 2007, Klein et al., 2009).

**Fig. 1:**
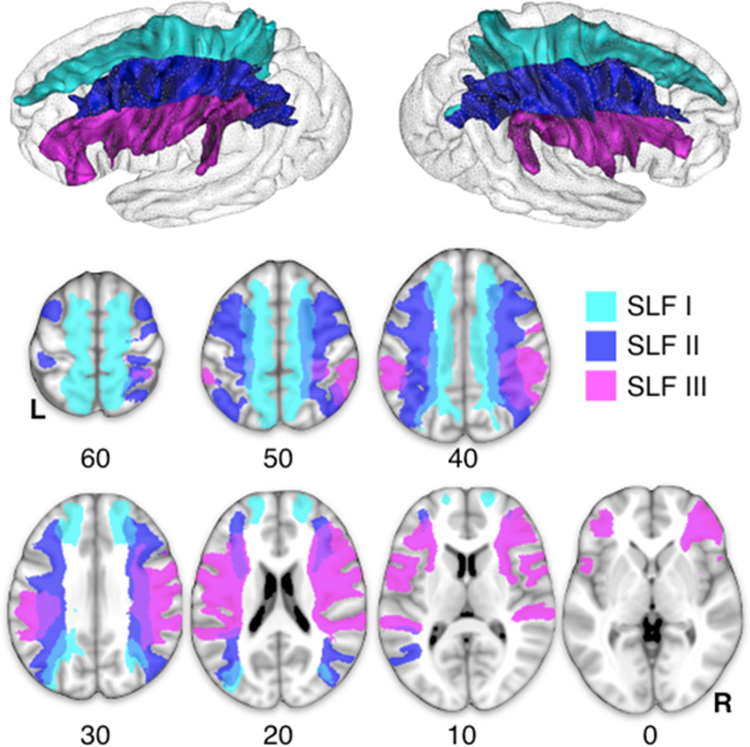
Mapping of the Superior Longitudinal Fasciculus (SLF). The top panel displays the average reconstruction of the SLF I (light blue), SLF II (navy blue) and SLF III (purple). The lower panel displays the axial sections of the three branches of the SLF. (For interpretation of the references to colour in this figure legend, the reader is referred to the web version of this article.)

Binary visitation maps were created for each tract by assigning each voxel a value of 1 or 0 depending on whether the voxel was intersected by the streamlines of the tract. Binary visitation maps of each dissected tracts were normalised to MNI space using the same affine with diffeomorphic deformations calculated above. We created percentage overlap maps using a previously published method (Thiebaut de Schotten et al., 2011b) by summing at each point in the MNI space the normalised visitation maps from each subject; hence the overlap of the visitation maps varied according to inter-subject variability. Fig. 1 displays the 3D rendering of the three SLFs onto the average rendering of the MNI152 template obtained using Anatomist 4.2 and BrainVISA 4.3 (http://brainvisa.info).

### 2.2 Meta-analyses

To obtain a comprehensive functional representation of the fronto-parietal networks we conducted 14 different meta-analyses of functions involving the co-activation of frontal and parietal regions. We first conducted a literature search in Pubmed (http://www.pubmed.com) of fMRI studies reporting fronto-parietal co-activations in healthy adults or adolescents, published between March 2002 and March 2012. We then selected papers including the keywords “frontal”, “parietal” and “fMRI”, and excluding the keywords “patients”, “disorders” and “connectivity” in their title or in their abstract. Among the selected 887 studies, we only considered those reporting frontal and parietal co-activations for the same contrast. In fact, compared to previous voxel-based meta-analyses (Radua et al., 2014), we did not aim to locate all the brain regions engaged during a given task but only fronto-parietal co-activations. To avoid biases towards liberally thresholded brain regions, we only selected contrasts reporting peak coordinates at the whole brain level in Montreal Neurological Institute space (MNI; www.mni.mcgill.ca) or Talairach space (Talairach and Tournoux, 1988). Studies including subjects taking medication or using any physiological manipulation paradigm (e.g. sleep deprivation) were excluded. Among the studies that fulfilled our selection criteria, 14 functions were identified as the most investigated and were selected for the current study. The list of the included studies and contrasts is reported in Supplementary Table 1.

These 14 functions included saccades, mental imagery (regrouping mental rotation and motor imagery), voluntary oriented and automatically captured attention, verbal and spatial working memory, phonological and semantic processing, motor sequences, response inhibition, number manipulation, emotion processing, decision making and mirror neuron-related functions (including action observation and theory of mind). Frontal and parietal peak coordinates from contrasts measuring the main or task-set effect of each function were collected, and a separate voxel-based meta-analysis was carried out for each of the 14 main functions using the Effect-Size Signed Differential Mapping software (www.sdmproject.com) (Radua and Mataix-Cols, 2012, Radua et al., 2012). First, a standard Talairach map of the effect-size of the regional activation was recreated separately for each study by means of a Gaussian kernel, which assigns higher effect-sizes to the voxels closer to peaks (with the effect size of the peaks being derived from the corresponding t-values). Second, the mean maps were calculated using standard meta-analytical random-effect models, which account for the variance and sample size of each study as well as for the between-study heterogeneity. Finally, statistical significance was assessed using a permutation test. Further details about this method are described in Radua and Mataix-Cols (2012) and Radua et al. (2012). Statistical maps were converted from Talairach space to MNI space using FMRIB’s Linear Image Registration Tool, provided with FSL. Cluster information is summarised in Supplementary Table 2.

### 2.3 Separate functional networks

In order to investigate the pattern of segregation of the 14 investigated functions, we first calculated cross correlations among the meta-analytic maps, using the function fslcc provided in the FSL software package, which were preliminary to the following principal component analysis (Fig. 3).

An ‘activation’ matrix was derived from the meta-analytic maps (Johansen-Berg et al., 2004). This matrix consisted of columns that indicated each meta-analytic map, and rows that represented the level of activation for each voxel in the frontal or parietal lobe. The correlation between the level of activation in each voxel for a certain function was correlated with the level of activation in the corresponding voxel for each of the other functions.

Further, the ‘activation’ matrix was entered into a first principal component analysis in SPSS (SPSS, Chicago, IL) using a covariance matrix and quartimax rotation (with a maximum of 50 iterations for convergence), in order to estimate the number of principal components to extract for each function (Fig. 4a). We plotted the components in order, according to their eigenvalue (y) and applied a scree test to separate the principal from residual components (Cattell, 1966). This first analysis revealed that two main factors were enough to explain more than 70% of the variance of the calculated meta-analytic maps.

A second principal component analysis was performed similarly, this time with a fixed number of two factors to extract. The result was used to group together meta-analytic maps sharing similar activations. A linear regression with 5.000 permutations, in which the weights of the raw components (i.e. the eigenvalues) represented the independent variable and the map of the functions the dependent variable, was run to detect brain regions having a statistically significant relationship with the two components. Results were Family Wise Error (FWE) corrected for multiple comparisons (p<0.05), and projected onto the average 3D rendering of the MNI152 template (Fig. 4).

### 2.4 Areas of shared activation

In order to reveal brain regions in the fronto-parietal cortex most likely to be recruited by all the 14 brain functions, we entered the meta-analytic maps into a one-sample *t*-test design with 5000 permutations. Statistics were FWE corrected for multiple comparisons. The result was projected onto the average 3D rendering of the MNI152 template (Fig. 5a).

### 2.5 White matter contribution to different functional networks

Finally, we quantified the contribution of the SLFs to the two identified groups of functions (Section 3), and to the areas of shared activation (Section 4). Average Z values of the functional maps were extracted at the location of the projections of the three branches of the SLF (with a 50% threshold). Results were reported in Figs. 5b and 6.

## 3 Results

### 3.1 Mapping of the SLF

The three branches of the SLF were identified in all the 129 healthy subjects by using spherical deconvolution tractography (Fig. 1).

### 3.2 Meta-analyses

**Fig. 2:**
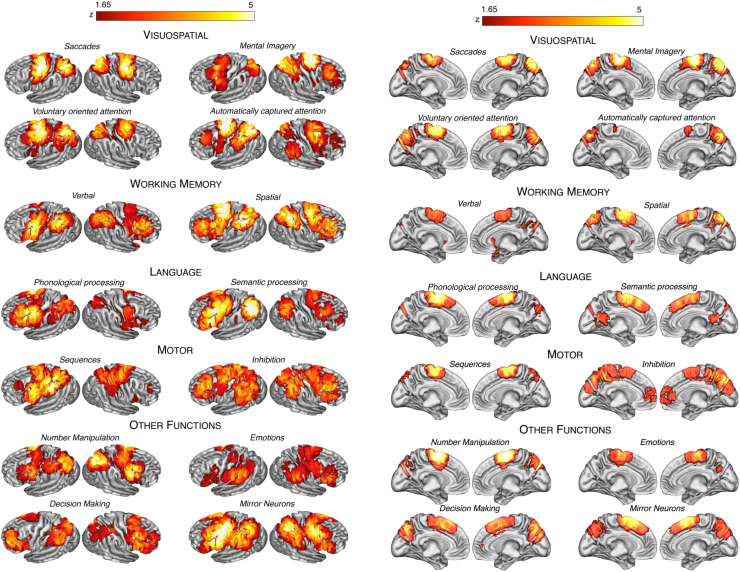
Meta-analytic maps. The maps of the 14 investigated functions are shown projected onto 3D-renderings of the brain (lateral and medial surfaces). A description of these maps can be found in Supplementary Results.

To obtain a comprehensive functional representation of the fronto-parietal networks we conducted 14 different meta-analyses of functions involving the co-activation of frontal and parietal regions (Fig. 2). Meta-analytic maps are described in Supplementary Results and are downloadable as Supplementary Material.

### 3.3 Separate functional networks

**Fig. 3:**
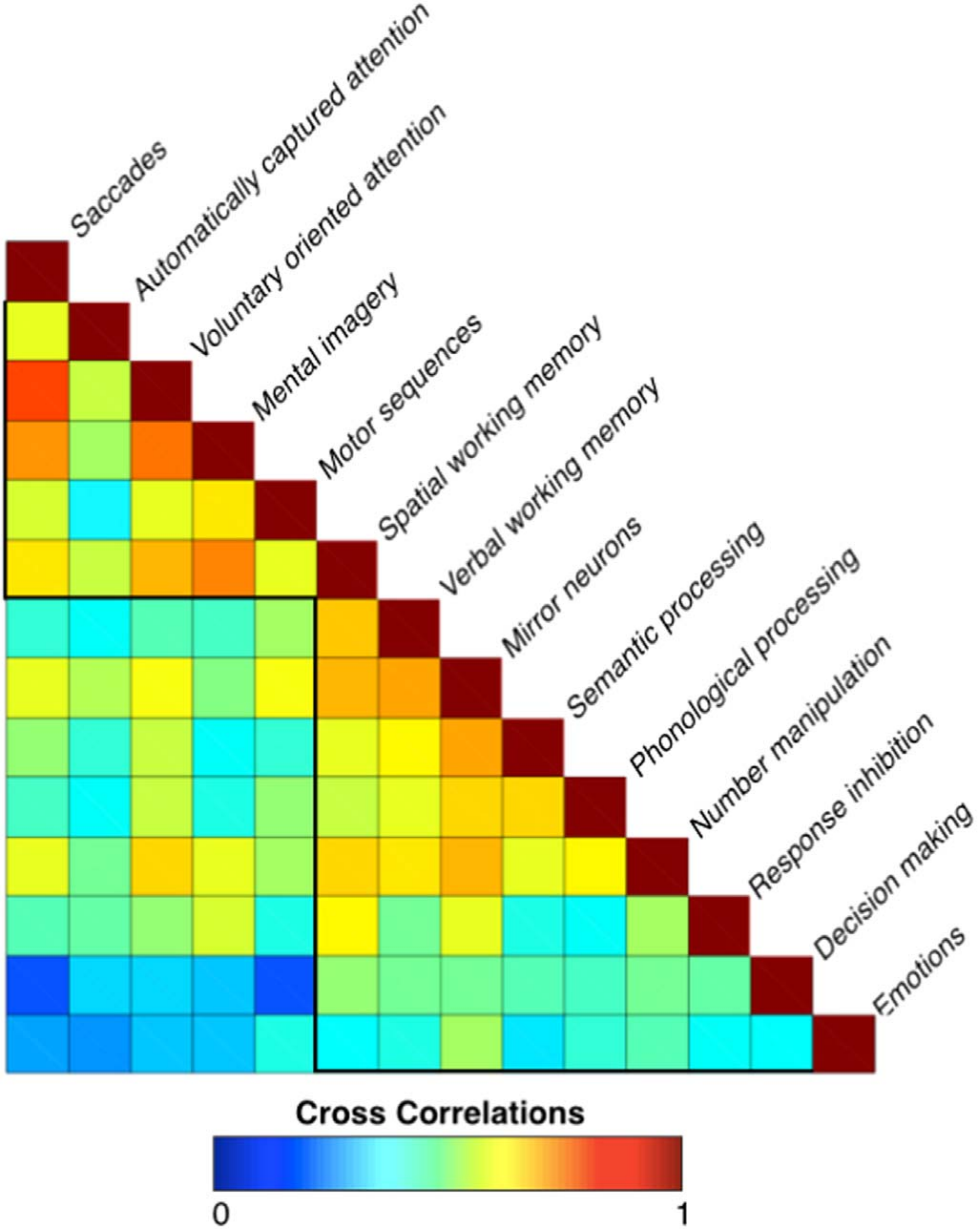
Cross-correlation. This panel displays the cross-correlations between the 14 meta-analytic maps. Two main clusters can be observed, one including spatial/motor functions and one including non-spatial/motor functions.

The cross correlation analysis of the meta-analytic maps revealed two clusters of highly correlated functions (Fig. 3). The first cluster included saccades, automatically captured and voluntary oriented attention, mental imagery, motor sequences and spatial working memory. The second comprised activations associated with verbal working memory, mirror neuron, semantic and phonological processing, number manipulation, response inhibition, decision-making and emotion processing. These findings suggest that the investigated fronto-parietal functions could be segregated into two groups, which are either involved or not in the manipulation of spatial/motor information.

**Fig. 4:**
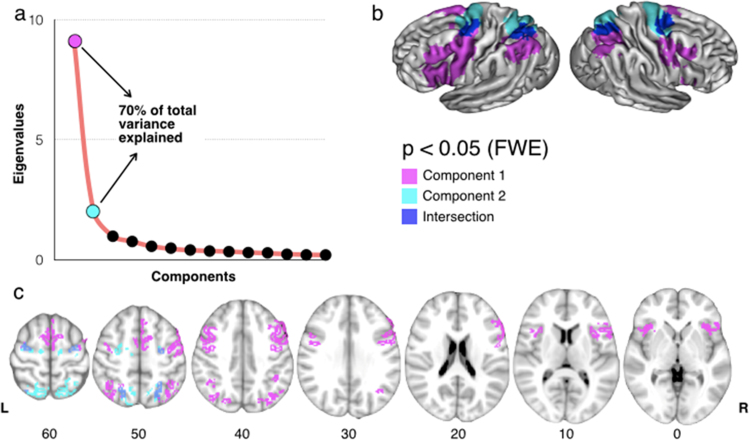
Principal component analysis. Panel ‘a’ shows the graph of the principal components (x) according to their eigenvalue sizes (y). Component 1 (pink) and component 2 (light blue) accounted for 70% of the total variance of the fronto-parietal activations. Panel ‘b’ and ‘c’ respectively show dorsolateral and medial tridimensional views and axial views of the two main components identified with the principal component analysis. Note that the intersection between the two components is displayed in dark blue. The raw weights for the different functions on the first two components are reported in Table 1. (For interpretation of the references to colour in this figure legend, the reader is referred to the web version of this article.)

To further confirm this segregation, a principal component analysis was carried out on the meta-analytic maps (Fig. 4). This revealed that only two principal components explain 70% of the total variance of the fronto-parietal co-activations. One of these components included saccades, voluntary oriented attention, mental imagery (regrouping motor imagery and mental rotation tasks) and motor sequences. As all these functions are involved in the processing of spatial/motor information, we included them under the ‘spatial/motor component’ umbrella. The other component comprised activations associated with working memory, mirror neurons, semantic and phonological processing, number manipulation, response inhibition, automatically captured attention, decision making and emotion processing. These functions were labelled as ‘non-spatial/motor’ in contrast to those included in the first component (Table 1). Therefore, the PCA segregated the 14 investigated functions into a spatial/motor and a non-spatial/motor component in agreement with the results of the cross-correlation analysis, with the exception of automatically captured attention and spatial working memory. However, the weights for these functions in the two components of the PCA were very similar (Table 1), suggesting that they may rely on both spatial/motor and non-spatial/motor information (please see Discussion for further comments). Notably, as shown in Fig. 4, the two components identified by the PCA were differently localised. The spatial/motor cluster mapped onto a dorsal fronto-parietal network connecting the superior parietal lobule to the posterior portion of the superior frontal gyrus. Conversely, the non-spatial/motor cluster mapped onto a ventral fronto-parietal network connecting the inferior parietal lobule to the inferior and middle frontal gyri.

### 3.4 Areas of shared activation

**Fig. 5:**
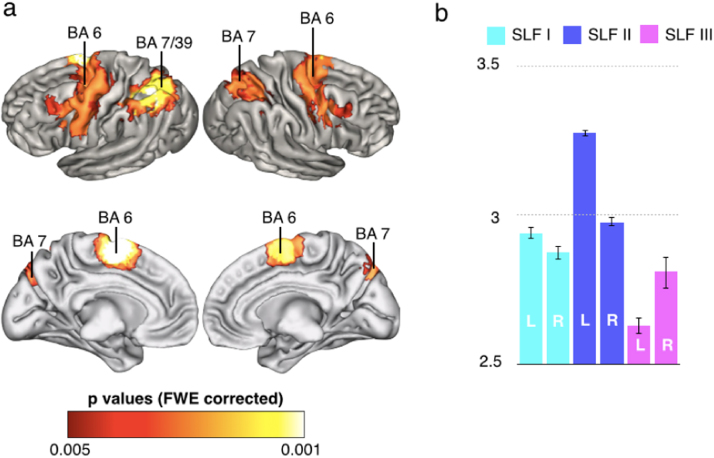
Areas of shared activation. Panel ‘a’ displays the map of fronto-parietal regions that are more probably activated by the 14 investigated functions (lateral and medial surfaces). BA: Brodmann area. Panel ‘b’ shows that the areas of shared activation are mostly associated with the SLF II. Average Z values of the functional maps at the location of the projections of the three branches of the SLF (with a 50% threshold) are reported. Error bars indicate confidence intervals (p<0.001).

In order to reveal brain regions in the fronto-parietal cortex most likely to be recruited by all the 14 brain functions, we entered the meta-analytic maps into a one-sample *t*-test design with 5000 permutations. We found that posterior frontal regions along the precentral gyrus and posterior parietal areas were significantly recruited by all the 14 functions (Fig. 5a). These areas included those at the intersection between the spatial/motor and non-spatial/motor networks described in Fig. 4b.

### 3.5 White matter contribution to different functional networks

**Fig. 6:**
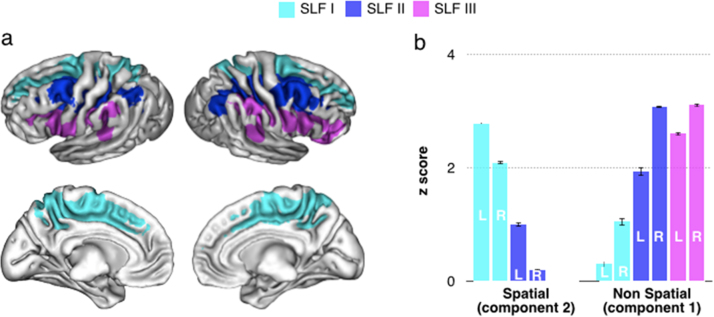
Functional roles of the Superior Longitudinal Fasciculus (SLF). Panel ‘a’ displays the cortical projections of the three branches of the SLF (lateral and medial view). Panel ‘b’ shows their functional correlates. We quantified the contribution of the SLFs to the spatial/motor and non-spatial/motor fronto-parietal meta-analytic maps. The SLF I appears to be primarily involved in spatial/motor functions, whereas the SLF III in non-spatial/motor functions. The SLF II was associated with both functions (see also Fig. 5b). Average Z values of the functional maps at the location of the projections of the three branches of the SLF (with a 50% threshold) are reported. Error bars indicate confidence intervals (p<0.001).

Finally, we quantified the contribution of the SLFs to the spatial/motor and non-spatial/motor fronto-parietal components (Section 3), and to the areas of shared activation (Section 4). We found that the SLF I represented the main tract underlying the spatial/motor cluster, whereas the SLF III was associated with the non-spatial/motor cluster (Fig. 6). The SLF II was associated with both functions and indeed, as shown in Fig. 5b, the areas of shared activation mostly corresponded to this tract (Fig. 5b).

## 4 Discussion

Our study presented novel findings on the functional organisation of the three branches of the SLF by combining tractography with a meta-analytic approach. Specifically, we found that the investigated fronto-parietal functions could be clustered into a dorsal network related to the manipulation of spatial/motor information and a ventral network dedicated to non-spatial/motor functions. Further, all 14 functions shared regions of activation located at the intersection of these two networks. Importantly, the dorsal and ventral networks were associated with different branches of the SLF. Indeed, the SLF I was the main tract associated with the spatial/motor cluster, whereas the SLF III underlay the non-spatial/motor cluster. Further, all the investigated functions activated a middle network mostly associated with the SLF II. In sum, our novel combined approach was successful in providing novel findings on the distinct functional roles of the three branches of the SLF, and can be applied to other white matter tracts.

The principal component analysis confirmed our hypothesis that fronto-parietal activations can be separated into a dorsal and a ventral component, and these in turn explain 70% of the total variance. Dorsal fronto-parietal areas were related to the manipulation of spatial/motor information, whilst ventral regions mainly supported non-spatial/motor functions. This result extended previous findings, which reported a dorso-ventral gradient between voluntary and reflexive saccadic movements (Mort et al., 2003), spatial and verbal working memory (Rottschy et al., 2012), and voluntary oriented and stimulus-grabbed visuo-spatial attention (Corbetta and Shulman, 2002). The PCA segregated the 14 investigated functions into a dorsal and a ventral component in agreement with the results of the cross-correlation analysis, except for visuo-spatial tasks requiring automatically captured attention and working memory. However, the weights for these functions in the two components of the PCA were very similar, suggesting that they may rely on both spatial and non-spatial information. Indeed, automatically captured attention is also involved in processing non-spatial aspects of a stimulus, such as its behavioural valence (Husain and Nachev, 2007, Corbetta and Shulman, 2011). Further, our meta-analysis and the work of others showed that spatial working memory relies on both fronto-parietal regions engaged independently of the type of stimuli, and more dorsal fronto-parietal areas devoted to the manipulation of their spatial content (Baddeley, 1986; Rottschy et al., 2012).

We also found that, even if very different from each other, the investigated functions were partly supported by shared fronto-parietal regions. The functional role of these shared regions is a matter of debate (Duncan, 2006, Dehaene and Changeux, 2011). For instance, according to the ‘multiple demand pattern model’, these regions constitute a core network that adapts to represent the information of many different tasks (Duncan, 2006). Our results show that the areas of shared activation include the supplementary motor area, inferior frontal sulcus, frontal operculum, and the intraparietal sulcus; and these have previously been identified as regions having very flexible response properties (Duncan and Owen, 2000). In agreement with this suggestion, single-cell recording studies in monkey carrying out a variety of tasks have shown that prefrontal cortex neurons flexibly code for the particular information that the current task requires (Everling et al., 2002, Everling et al., 2006).

Alternatively, shared activations may represent part of the network that mediates a modality-independent conscious access (Dehaene and Changeux, 2011). This model supports a two-stage processing of sensory information. The initial stage involves parallel and non-conscious perception of sensory stimuli, followed by occasional access to a secondary, serial conscious processing of individual or integrated information. The latter stage relies on a common network that has been located in the fronto-parietal cortex (Pashler, 1994). The areas of shared activation we report may thus represents the final relay of an obligatory passage of information from a non-conscious to a conscious level. Indeed, experimental evidence suggests that transcranial magnetic stimulation of the frontal or parietal areas that form this shared network (Kanai et al., 2008, Quentin et al., 2015, Quentin et al., 2016), or lesions to its connections (Thiebaut de Schotten, 2014), modify conscious perception. Hence, taken together, our work and that of others suggests that the two models are closely linked and could be integrated in a unitary explanation of the core fronto-parietal functions shared by spatial and non-spatial tasks.

We also demonstrated that different branches of the SLF support the functional segregation between dorsal and ventral fronto-parietal networks, as well as their integration. Overall, our results support the conclusion that the SLF I is primarily associated with spatial/motor functions, whereas the SLF III with non-spatial/motor functions. Regions of shared activation are mainly associated with the SLF II. Anatomically the SLF II projects from ventral parietal regions to dorsal frontal regions and may therefore represent a bridge between the dorsal and ventral fronto-parietal networks. This interpretation is supported by previous studies in which damage to the SLF II has been associated with both spatial and non-spatial deficits in hemispatial neglect (Husain and Rorden, 2003, Bartolomeo et al., 2007).

Taken together, our findings support our hypothesis that the SLF branches are associated with different functional roles. This kind of functional investigations are particularly relevant as they may help support the appropriateness of the anatomical subdivision of the SLF itself, considering that the number of its components in the human brain is still debated (Thiebaut de Schotten et al., 2011a, Thiebaut de Schotten et al., 2011b; Martino and De Lucas, 2014; Wang et al., 2015). First of all, early anatomists used the terms SLF and arcuate fasciculus as synonyms and, despite axonal tracing studies (Petrides and Pandya, 1984) and electrophysiological techniques (Rizzolatti at al., 1998) showed that a group of fronto-parietal fibres (SLF) can be separated by those arching around the Sylvian fissure (long segment of the arcuate fasciculus), confusion of terminology has remained in human studies where these techniques cannot be used. Indeed, the large majority of tractography studies considered the SLF as a single bundle often not clearly separated by the arcuate fasciculus (Broser et al., 2012, Agosta et al., 2013, Myall et al., 2013, Abhinav et al., 2014, Kamali et al., 2014, Meyer et al., 2014). Only recent advances in tractography enabled the visualisation of the entire anatomy of the SLF crossing through the corticospinal tract and thus its separation from the arcuate and subdivision into distinct components (Makris et al., 2005; Thiebaut de Schotten et al., 2011a, Thiebaut de Schotten et al., 2011b; Catani et al., 2012; Chechlacz et al., 2015; Marshall et al., 2015; Wang et al., 2015; Budisavljevic et al., 2016; Cazzoli and Chechlacz, 2016). Nevertheless, there is no consensus on the most appropriate subdivision. For instance, beyond the model used in the current investigation, which separates the SLF in three branches along the dorso-ventral axis (Makris et al., 2005; Thiebaut de Schotten et al., 2011a, Thiebaut de Schotten et al., 2011b), it has been proposed that the SLF is composed of three perisylvian branches (which correspond to the long, posterior and anterior segment of the arcuate fasciculus) and two non-perisylvian branches (which correspond to the SLF I and II) (Martino and De Lucas, 2014). Further, the existence of the dorsal branch (SLF I) has been challenged by a DSI and anatomical study, which reported that the SLF I could not be consistently reconstructed in healthy subjects or identified through anatomical dissections (Wang et al., 2015). The authors concluded that the SLF should be subdivided in a dorsal (SLF II) and a ventral (SLF III) component, whereas the SLF I should be considered part of the cingulum system. This result contrasts with other studies combining tractography and anatomical dissections (Thiebaut de Schotten et al., 2011a, Thiebaut de Schotten et al., 2011b; Yagmurlu et al., 2015). For instance, the latter reconstructed the three branches of the SLF in 50 human hemispheres and concluded that the SLF I has a close anatomical relationship with the cingulum but it does not reach it, as it runs above the cingulate sulcus. Inconsistencies among studies may be related to methodological differences or individual variability. Functional investigations as the current one may positively contribute to this debate on the subdivision of the SLF by providing information on the different functions supported by its distinct components.

In addition to the described dorso-ventral gradient, the distribution of our spatial/motor and non-spatial/motor components (Fig. 4b) suggests that functional activations might also reflect a central-to-peripheral gradient centred around the primary motor-sensory cortex. This gradient indicates that spatial somatosensory-motor control may be supported by more central areas, in the precentral and postcentral gyri, whereas more abstract functions, such as decision making, may involve peripheral fronto-parietal regions more extensively. Indeed, this observation is supported by the cross-correlation, which showed that motor coordination and saccades are the functions least correlated with decision making. The existence of this gradient along the rostro-caudal axis is in agreement with previous neurodevelopmental (Zhan et al., 2013), functional (Koechlin et al., 2003, Badre and D’Esposito, 2007, Badre and D’Esposito, 2009), and anatomical studies (Thiebaut de Schotten et al., 2016), and provides further insight into the functional organisation of the frontal-parietal networks.

Finally, although the current study benefitted from a very powerful meta-analytical approach, there are also some limitations that need to be acknowledged. Our combined approach offered the advantage of exploring several functions at the same time, and of being independent from an *a priori* hypothesis regarding tract functions. However, the meta-analytic maps were normalised and compared with the tractography reconstruction of white matter tracts derived from a different dataset. Hence, functional activation of cortical regions and structural information on white matter anatomy were based on two distinct populations, which limited our ability to take into account inter-individual variability of the real anatomy. Future validation studies of our results are therefore needed using both tractography and fMRI obtained from the same subjects in order to quantify the relationship between anatomical (i.e. microstructure or volume of the tracts), behavioural (i.e. test performance or observed pathology) and functional (i.e. level or localisation of the activation) variables (Thiebaut de Schotten et al., 2014). Tractography and fMRI data obtained from the same subjects is also required to clarify whether the SLF branches are involved in different functions in the left and the right hemisphere. Secondly, we estimated the cortical projections of the SLF I, II and III using tractography. Although our result is consistent with previous axonal tracing studies (Schmahmann and Pandya, 2006), projections to the gyrus walls may have been underestimated due to tractography limitations. New algorithms modelling the fanning of tractography endpoints should be the subject of further research (Van Essen et al., 2014). Thirdly, we focused our analysis on 14 most investigated fronto-parietal functions. This decision was dictated by the number of published papers, as we needed to have a minimum number of articles for function in order to reliably perform a meta-analysis. All included functions have at least 7 papers available and responding to the selection criteria detailed in Methods. We are aware that other functions may involve fronto-parietal co-activations but they received less attention in the literature and could not be considered for this study. For instance, our analysis of motor functions was mainly limited to voluntary saccades and finger tapping tasks, as these are the paradigms most feasible and commonly used in fMRI studies (Witt et al., 2008). We found that these tasks more consistently elicited the activation of dorsal fronto-parietal areas, and were therefore associated with the SLF I. This association captured the recruitment of more dorsal regions of the motor homunculus, but also the involvement of brain areas coding for the spatial aspects underlying movements (Gullivan and Culham, 2015). However, ventral fronto-parietal areas may contribute to motor performance for instance during hand-mouth co-ordination (Yokochi et al., 2003) and tool making or use (Hecht et al., 2015; Martin et al., 2016). Nevertheless, we could not rule out the potential role of ventral connections, such s the SLF III, in the control of these more complex motor tasks due to the limited role of fMRI studies in their investigation. Similarly, the tasks included in our meta-analyses involved the manipulation of stimuli in the space that directly surrounds the subject, i.e. those located in the peripersonal space. This has been reported to elicit the activation of more dorsal regions as compared to the manipulation of stimuli in the far (extrapersonal) space (Bjoertomt et al., 2002, 2009), but the latter is not equally testable inside the MRI scanner. Also, we focused on fronto-parietal regions but many of the 14 functions we analysed rely on more extended networks. Hence, our results should not be considered as comprehensive of the whole functional networks associated with specific tasks. Further, three meta-analyses included a paper with a sample of adolescents, whose pattern of activation may be similar but not identical to that of adults. However, the use of a meta-analytic approach to define the areas more consistently activated during a task guaranteed that only those commonly activated by adults and adolescents were considered for the following analyses. Finally, although a SDM meta-analysis represents a substantial advance for the integration of functional neuroimaging data, all meta-analytic methods have a number of limitations, such as publication bias, which should be considered when interpreting the final results (Jennings and Van Horn, 2012).

In conclusion, 10 years of fMRI studies combined with advanced diffusion tractography suggest that fronto-parietal functions can be segregated into dorsal spatial/motor and ventral non-spatial/motor networks, which respectively overlap with the projections of the SLF I and SLF III. The SLF II corresponds to a network of multimodal region at the intersection between the dorsal and ventral networks. The regions connected by the SLF II may host neurons with very flexible response properties and embody our conscious processing. Our novel combined approach was successful in providing novel findings on the distinct functional roles of the three branches of the SLF, and can be applied to other white matter tracts.

## Conflict of interest

The authors declare no competing financial interests.

## Figures and Tables

**Table 1:** Principal component analysis. The table reports the raw weights for the different functions on the first two components identified by the PCA. As shown, the first 10 functions have higher weights for the first component (non-spatial/motor), whereas the last 4 have higher weights for the second component (spatial/motor).

**Function**	**Component 1**	**Component 2**
Mirror neurons	1.258	.142
Semantic processing	.970	.018
Verbal working memory	.907	.054
Phonological processing	.740	.023
Decision making	.461	−.073
Number manipulation	.785	.349
Emotion processing	.367	−.013
Response Inhibition	.581	.355
Spatial working memory	.903	.742
Involuntary captured attention	.373	.316
Mental Imagery	.438	1.155
Saccades	.481	.866
Voluntary oriented attention	.558	.786
Motor sequences	.507	.544
