# Supplementary material for: Functional segregation and integration within fronto-parietal networks
Source: Neuroimage. 2017 Feb 1;146:367–75. doi: 10.1016/j.neuroimage.2016.08.031 (PMC5312783; doi:10.1016/j.neuroimage.2016.08.031)
Supplement: Supplementary file 4 — Supplementary material [file mmc4.pdf]

SUPPLEMENTARY INFORMATION

SUPPLEMENTARY RESULTS

META-ANALYSES

1.Visuo-spatial functions

Meta-analytic maps (Figure 1) showed that voluntary oriented attention mainly recruited dorsal areas, such as the intraparietal sulcus/superior parietal lobule and the frontal eye field (FEF), whereas automatic captured attention extended to more ventral regions such as the middle frontal and the angular gyri. In other words, they supported that voluntary oriented and automatically captured visuo-spatial attention are partially segregated functions (Corbetta and Shulman, 2002; Thiebaut de Schotten et al., 2011), in agreement with convergent results coming from PET studies (Corbetta et al., 1993; Nobre et al., 1997), *f*MRI data (Corbetta et al., 2000; Hopfinger et al., 2000) and functional connectivity analyses (Fox et al., 2006; He et al., 2007). Notably, the nodes of the dorsal attentive network were consistently recruited during voluntary saccadic movements, as in previous simian and human reports. For instance, the microstimulation of the FEF in the simian brain affects both saccadic movements and the performance in attention-demanding tasks (Moore and Fallah, 2001), whilst functional imaging studies report a strong overlap between covert spatial attention and overt oculomotor shifts in the nodes of the dorsal network (Corbetta, 1998; Nobre et al., 2000). Mental imagery, regrouping motor imagery and mental rotation tasks, consistently activated the FEF, the mid portion of the middle frontal gyrus, the intraparietal sulcus and the superior parietal lobule, suggesting that both these tasks rely on the same fronto-parietal regions (Hanakawa et al., 2003; Lotze and Halsband, 2006; Mourao-Miranda et al., 2009; Vry et al., 2012). Specifically, it has been reported that the prefrontal cortex modulates the activation of primary areas in a content-selective manner, whereas the parietal

cortex may contribute to the generation of mental images regardless of their content, probably through the engagement of attentive resources (Mechelli et al., 2004). Ventral fronto-parietal areas appear to contribute with less consistency, contrasting with a previous report (Vry et al., 2012), probably in relation to the different tasks included.

2.Working Memory

The segregated localisation and the hemispheric lateralisation of verbal and spatial working memory is the matter of debate (Smith and Jonides, 1998; Walter et al., 2003; Owen et al., 2005). Our meta-analyses revealed that verbal and spatial working memory shared bilateral activations in the posterior middle and inferior frontal gyri, the supplementary motor area, the supramarginal and angular gyri. However, spatial working memory tasks specifically recruited more dorsal areas, such as the FEF, the anterior portion of the middle frontal gyrus and the superior parietal lobule, whereas verbal working memory engaged more ventral regions, such as Broca's territory and the inferior parietal lobule. Taken together, these findings support the existence of a "core" bilateral network engaged by working memory independently of the type of stimuli, task or contrast (Baddeley, 1986; Rottschy et al., 2012), and of a dorso-ventral gradient between areas respectively devoted to spatial or verbal working memory contents.

3.Language

Linguistic functions were clearly left-lateralised and showed a partial segregation among the areas dedicated to either phonological or semantic processing. Both functions co-activated Broca's and supplementary motor areas. Phonological processing also activated the supramarginal gyrus, whereas semantic processing specifically recruited the posterior middle frontal and the angular gyri. This is in accordance with previous reports (Vigneau et al., 2006; Price, 2012).

4.Motor functions

Motor coordination activated motor and somatosensory areas but also the supramarginal gyrus and the anterior portion of the superior parietal lobule, in agreement with a previous meta-analysis (Witt et al., 2008). The inclusion of several tasks employing the right dominant hand explains the greater consistency in the left hemisphere, whereas the use of complex finger sequences may induce the observed consistent recruitment of the posterior parietal cortex, which retrieves memorised sequences to translate them into plans of execution (Sakai et al., 1998; Shannon and Buckner, 2004). Conversely, motor inhibition tasks elicited a more extended activation of fronto-parietal regions, including the anterior and posterior portion of the middle frontal gyrus, posterior portion of the superior frontal gyrus, medial precentral gyrus and the whole parietal lobe. The general pattern of activation is in agreement with previous meta-analyses (Swick et al., 2011; Criaud and Boulinguez, 2013), whereas the global low level of consistency may be explained by the inclusion of different response inhibition tasks. Swick and colleagues (2011) have in fact reported that the widely used Go/No Go Task and the Stop Signal Task actually produce overlapping as well as different regions of activation (Swick et al., 2011). The former engages the fronto-parietal network to a greater extent than the latter, which is mainly supported by a cingulo-opercular network. The extensive activation, as well as the differences reported for these two tasks, suggests that response inhibition is a multifaceted process, whose different components are supported by dissociable neural systems (Burnett Heyes et al., 2012). Moreover, fronto-parietal activations have been associated with task complexity and argued to reflect the employment of attentive and working memory resources, instead of motor inhibition *per se* (Simmonds et al., 2008; Hampshire et al., 2010; Criaud and Boulinguez, 2013).

5.Other functions

Number manipulation more specifically activated the angular gyrus and the superior parietal lobule, extending to the supramarginal gyrus and the posterior portions of the superior,

middle and inferior frontal gyri. This fronto-parietal activation is consistent with a recent meta-analysis including 53 studies of number and calculation tasks (Arsalidou and Taylor, 2011), which found that the recruitment of the frontal lobe and the pattern of lateralisation are related to calculation complexity. Both this study and our findings support the need of adding a frontal component to the classical tripartite model proposed by Dehaene and co-workers (Dehaene et al., 2003). The level of concordance in the areas activated by decision-making tasks is low, indicating a low reproducibility across studies. However, we identified bilateral frontal areas, such as the most anterior portion of the middle frontal gyrus and the inferior frontal gyrus (extending towards the frontal pole and the orbitofrontal cortex) and the supplementary motor area, as the regions possibly hosting the core of the decision making process, in agreement with previous literature reports (Brand et al., 2006; Koechlin and Hyafil, 2007; Heekeren et al., 2008; Kouneiher et al., 2009; Summerfield and Koechlin, 2010; Rushworth et al., 2011). The supramarginal, angular and superior parietal lobule are also involved. The inferior frontal/orbitofrontal cortex, medial SFG and the supramarginal gyrus were significantly activated during emotion processing, as previously reported by studies investigating emotional face processing and affective prosody (Phan et al., 2002; Wildgruber et al., 2005; Fusar-Poli et al., 2009; Bruck et al., 2011). Bruck et al. (2011) suggested that fronto-parietal areas are recruited for the cognitive evaluation of emotional percepts occurring after the extraction and integration of acoustic and visual features (Bruck et al., 2011). Mirror neurons are considered to be very responsive to the representation of other's motor action, thoughts and emotions by the corresponding processes in oneself, and thus to be the core mechanism underlying imitation, theory of mind and empathy (Gallese and Goldman, 1998; Rizzolatti et al., 2001; Preston and de Waal, 2002). In agreement with previous studies (Rizzolatti and Arbib, 1998; Buccino et al., 2001; Buccino et al., 2004), we found that mirror neurons tasks recruited the precentral gyrus, extending in the frontal lobe to the posterior portion of the middle

and inferior frontal gyri and, in the parietal lobe, to the supplementary motor area, supramarginal and angular gyri and superior parietal lobule.

SUPPLEMENTARY REFERENCES

1.Articles cited in Supplementary Results

Arsalidou M, Taylor MJ (2011) Is 2+2=4? Meta-analyses of brain areas needed for numbers and calculations. NeuroImage 54:2382-2393.

Baddeley AD (1986) Working memory. Oxford Oxfordshire, New York: Clarendon Press;Oxford University Press.

Brand M, Labudda K, Markowitsch HJ (2006) Neuropsychological correlates of decision-making in ambiguous and risky situations. Neural networks : the official journal of the International Neural Network Society 19:1266-1276.

Bruck C, Kreifelts B, Wildgruber D (2011) Emotional voices in context: a neurobiological model of multimodal affective information processing. Physics of life reviews 8:383-403.

Buccino G, Binkofski F, Riggio L (2004) The mirror neuron system and action recognition. Brain and language 89:370-376.

Buccino G, Binkofski F, Fink GR, Fadiga L, Fogassi L, Gallese V, Seitz RJ, Zilles K, Rizzolatti G, Freund HJ (2001) Action observation activates premotor and parietal areas in a somatotopic manner: an fMRI study. The European journal of neuroscience 13:400-404.

Burnett Heyes S, Adam RJ, Urner M, van der Leer L, Bahrami B, Bays PM, Husain M (2012) Impulsivity and rapid decision-making for reward. Frontiers in psychology 3:153.

Corbetta M (1998) Frontoparietal cortical networks for directing attention and the eye to visual locations: identical, independent, or overlapping neural systems? Proceedings of the National Academy of Sciences of the United States of America 95:831-838.

Corbetta M, Shulman GL (2002) Control of goal-directed and stimulus-driven attention in the brain. Nature reviews Neuroscience 3:201-215.

Corbetta M, Miezin FM, Shulman GL, Petersen SE (1993) A PET study of visuospatial attention. The Journal of neuroscience : the official journal of the Society for Neuroscience 13:1202-1226.

Corbetta M, Kincade JM, Ollinger JM, McAvoy MP, Shulman GL (2000) Voluntary orienting is dissociated from target detection in human posterior parietal cortex. Nature neuroscience 3:292-297.

Criaud M, Boulinguez P (2013) Have we been asking the right questions when assessing response inhibition in go/no-go tasks with fMRI? A meta-analysis and critical review. Neuroscience and biobehavioral reviews 37:11-23.

Dehaene S, Piazza M, Pinel P, Cohen L (2003) Three parietal circuits for number processing. Cognitive neuropsychology 20:487-506.

Fox MD, Corbetta M, Snyder AZ, Vincent JL, Raichle ME (2006) Spontaneous neuronal activity distinguishes human dorsal and ventral attention systems. Proceedings of the National Academy of Sciences of the United States of America 103:10046-10051.

Fusar-Poli P, Placentino A, Carletti F, Landi P, Allen P, Surguladze S, Benedetti F, Abbamonte M, Gasparotti R, Barale F, Perez J, McGuire P, Politi P (2009) Functional atlas of emotional faces processing: a voxel-based meta-analysis of 105 functional magnetic resonance imaging studies. Journal of psychiatry & neuroscience : JPN 34:418-432.

Gallese V, Goldman A (1998) Mirror neurons and the simulation theory of mind-reading. Trends in cognitive sciences 2:493-501.

Hampshire A, Chamberlain SR, Monti MM, Duncan J, Owen AM (2010) The role of the right inferior frontal gyrus: inhibition and attentional control. NeuroImage 50:1313-1319.

Hanakawa T, Immisch I, Toma K, Dimyan MA, Van Gelderen P, Hallett M (2003) Functional properties of brain areas associated with motor execution and imagery. Journal of neurophysiology 89:989-1002.

He BJ, Snyder AZ, Vincent JL, Epstein A, Shulman GL, Corbetta M (2007) Breakdown of functional connectivity in frontoparietal networks underlies behavioral deficits in spatial neglect. Neuron 53:905-918.

Heekeren HR, Marrett S, Ungerleider LG (2008) The neural systems that mediate human perceptual decision making. Nature reviews Neuroscience 9:467-479.

Hopfinger JB, Buonocore MH, Mangun GR (2000) The neural mechanisms of top-down attentional control. Nature neuroscience 3:284-291.

Koechlin E, Hyafil A (2007) Anterior prefrontal function and the limits of human decision-making. Science 318:594-598.

Kouneiher F, Charron S, Koechlin E (2009) Motivation and cognitive control in the human prefrontal cortex. Nature neuroscience 12:939-945.

Lotze M, Halsband U (2006) Motor imagery. Journal of physiology, Paris 99:386-395.

Mechelli A, Price CJ, Friston KJ, Ishai A (2004) Where bottom-up meets top-down: neuronal interactions during perception and imagery. Cereb Cortex 14:1256-1265.

Moore T, Fallah M (2001) Control of eye movements and spatial attention. Proceedings of the National Academy of Sciences of the United States of America 98:1273-1276.

Mourao-Miranda J, Ecker C, Sato JR, Brammer M (2009) Dynamic changes in the mental rotation network revealed by pattern recognition analysis of fMRI data. Journal of cognitive neuroscience 21:890-904.

Nobre AC, Gitelman DR, Dias EC, Mesulam MM (2000) Covert visual spatial orienting and saccades: overlapping neural systems. NeuroImage 11:210-216.

Nobre AC, Sebestyen GN, Gitelman DR, Mesulam MM, Frackowiak RS, Frith CD (1997) Functional localization of the system for visuospatial attention using positron emission tomography. Brain : a journal of neurology 120 (Pt 3):515-533.

Owen AM, McMillan KM, Laird AR, Bullmore E (2005) N-back working memory paradigm: a meta-analysis of normative functional neuroimaging studies. Human brain mapping 25:46-59.

Phan KL, Wager T, Taylor SF, Liberzon I (2002) Functional neuroanatomy of emotion: a meta-analysis of emotion activation studies in PET and fMRI. NeuroImage 16:331-348.

Preston SD, de Waal FB (2002) Empathy: Its ultimate and proximate bases. The Behavioral and brain sciences 25:1-20; discussion 20-71.

Price CJ (2012) A review and synthesis of the first 20years of PET and fMRI studies of heard speech, spoken language and reading. NeuroImage 62:816-847.

Rizzolatti G, Arbib MA (1998) Language within our grasp. Trends in neurosciences 21:188-194.

Rizzolatti G, Fogassi L, Gallese V (2001) Neurophysiological mechanisms underlying the understanding and imitation of action. Nature reviews Neuroscience 2:661-670.

Rottschy C, Langner R, Dogan I, Reetz K, Laird AR, Schulz JB, Fox PT, Eickhoff SB (2012) Modelling neural correlates of working memory: a coordinate-based meta-analysis. NeuroImage 60:830-846.

Rushworth MF, Noonan MP, Boorman ED, Walton ME, Behrens TE (2011) Frontal cortex and reward-guided learning and decision-making. Neuron 70:1054-1069.

Sakai K, Hikosaka O, Miyauchi S, Takino R, Sasaki Y, Putz B (1998) Transition of brain activation from frontal to parietal areas in visuomotor sequence learning. The Journal of neuroscience : the official journal of the Society for Neuroscience 18:1827-1840.

Shannon BJ, Buckner RL (2004) Functional-anatomic correlates of memory retrieval that suggest nontraditional processing roles for multiple distinct regions within posterior parietal cortex. The Journal of neuroscience : the official journal of the Society for Neuroscience 24:10084-10092.

Simmonds DJ, Pekar JJ, Mostofsky SH (2008) Meta-analysis of Go/No-go tasks demonstrating that fMRI activation associated with response inhibition is task-dependent. Neuropsychologia 46:224-232.

Smith EE, Jonides J (1998) Neuroimaging analyses of human working memory. Proceedings of the National Academy of Sciences of the United States of America 95:12061-12068.

Summerfield C, Koechlin E (2010) Economic value biases uncertain perceptual choices in the parietal and prefrontal cortices. Frontiers in human neuroscience 4:208.

Swick D, Ashley V, Turken U (2011) Are the neural correlates of stopping and not going identical? Quantitative meta-analysis of two response inhibition tasks. NeuroImage 56:1655-1665.

Thiebaut de Schotten M, Dell'Acqua F, Forkel SJ, Simmons A, Vergani F, Murphy DG, Catani M (2011) A lateralized brain network for visuospatial attention. Nature neuroscience 14:1245-1246.

Vigneau M, Beaucousin V, Herve PY, Duffau H, Crivello F, Houde O, Mazoyer B, Tzourio-Mazoyer N (2006) Meta-analyzing left hemisphere language areas: phonology, semantics, and sentence processing. NeuroImage 30:1414-1432.

Vry MS, Saur D, Rijntjes M, Umarova R, Kellmeyer P, Schnell S, Glauche V, Hamzei F, Weiller C (2012) Ventral and dorsal fiber systems for imagined and executed movement. Experimental brain research Experimentelle Hirnforschung Experimentation cerebrale 219:203-216.

Walter H, Bretschneider V, Gron G, Zurowski B, Wunderlich AP, Tomczak R, Spitzer M (2003) Evidence for quantitative domain dominance for verbal and spatial working memory in

frontal and parietal cortex. Cortex; a journal devoted to the study of the nervous system and behavior 39:897-911.

Wildgruber D, Riecker A, Hertrich I, Erb M, Grodd W, Ethofer T, Ackermann H (2005) Identification of emotional intonation evaluated by fMRI. NeuroImage 24:1233-1241.

Witt ST, Laird AR, Meyerand ME (2008) Functional neuroimaging correlates of finger-tapping task variations: an ALE meta-analysis. NeuroImage 42:343-356.

<u>2. Articles included in the meta-analyses</u>

Abd Hamid AI, Yusoff AN, Mukari SZ, Mohamad M (2011) Brain Activation during Addition and Subtraction Tasks In-Noise and In-Quiet. The Malaysian journal of medical sciences : MJMS 18:3-15.

Addicott MA, Baranger DA, Kozink RV, Smoski MJ, Dichter GS, McClernon FJ (2012) Smoking withdrawal is associated with increases in brain activation during decision making and reward anticipation: a preliminary study. Psychopharmacology 219:563-573.

Albouy G, Sterpenich V, Vandewalle G, Darsaud A, Gais S, Rauchs G, Desseilles M, Boly M, Dang-Vu T, Balteau E, Degueldre C, Phillips C, Luxen A, Maquet P (2012) Neural correlates of performance variability during motor sequence acquisition. NeuroImage 60:324-331.

Alvarez TL, Alkan Y, Gohel S, Douglas Ward B, Biswal BB (2010) Functional anatomy of predictive vergence and saccade eye movements in humans: a functional MRI investigation. Vision research 50:2163-2175.

Asahi S, Okamoto Y, Okada G, Yamawaki S, Yokota N (2004) Negative correlation between right prefrontal activity during response inhibition and impulsiveness: a fMRI study. European archives of psychiatry and clinical neuroscience 254:245-251.

Bach P, Peelen MV, Tipper SP (2010) On the role of object information in action observation: an fMRI study. Cereb Cortex 20:2798-2809.

Binder JR, Medler DA, Desai R, Conant LL, Liebenthal E (2005) Some neurophysiological constraints on models of word naming. NeuroImage 27:677-693.

Boecker H, Jankowski J, Ditter P, Scheef L (2008) A role of the basal ganglia and midbrain nuclei for initiation of motor sequences. NeuroImage 39:1356-1369.

Boehler CN, Appelbaum LG, Krebs RM, Chen LC, Woldorff MG (2011) The role of stimulus salience and attentional capture across the neural hierarchy in a stop-signal task. PloS one 6:e26386.

Bolster RB, D'Arcy RC, Song X, Runke DS, Ryner L (2011) Detection versus location judgments in a hidden pattern task: functional MRI and behavioral correlates. Journal of clinical and experimental neuropsychology 33:765-775.

Braet W, Johnson KA, Tobin CT, Acheson R, Bellgrove MA, Robertson IH, Garavan H (2009) Functional developmental changes underlying response inhibition and error-detection processes. Neuropsychologia 47:3143-3151.

Braet W, Johnson KA, Tobin CT, Acheson R, McDonnell C, Hawi Z, Barry E, Mulligan A, Gill M, Bellgrove MA, Robertson IH, Garavan H (2011) fMRI activation during response inhibition and error processing: the role of the DAT1 gene in typically developing adolescents and those diagnosed with ADHD. Neuropsychologia 49:1641-1650.

Buccino G, Binkofski F, Riggio L (2004a) The mirror neuron system and action recognition. Brain and language 89:370-376.

Buccino G, Lui F, Canessa N, Patteri I, Lagravinese G, Benuzzi F, Porro CA, Rizzolatti G (2004b) Neural circuits involved in the recognition of actions performed by nonconspecifics: an FMRI study. Journal of cognitive neuroscience 16:114-126.

Calvert GA, Campbell R (2003) Reading speech from still and moving faces: the neural substrates of visible speech. Journal of cognitive neuroscience 15:57-70.

Camchong J, Dyckman KA, Chapman CE, Yanasak NE, McDowell JE (2006) Basal ganglia-thalamocortical circuitry disruptions in schizophrenia during delayed response tasks. Biological psychiatry 60:235-241.

Canessa N, Gorini A, Cappa SF, Piattelli-Palmarini M, Danna M, Fazio F, Perani D (2005) The effect of social content on deductive reasoning: an fMRI study. Human brain mapping 26:30-43.

Cazzato V, Macaluso E, Crostella F, Aglioti SM (2012) Mapping reflexive shifts of attention in eye-centered and hand-centered coordinate systems. Human brain mapping 33:165-178.

Chan RC, Rao H, Chen EE, Ye B, Zhang C (2006) The neural basis of motor sequencing: an fMRI study of healthy subjects. Neuroscience letters 398:189-194.

Chen SH, Desmond JE (2005a) Temporal dynamics of cerebro-cerebellar network recruitment during a cognitive task. Neuropsychologia 43:1227-1237.

Chen SH, Desmond JE (2005b) Cerebrocerebellar networks during articulatory rehearsal and verbal working memory tasks. NeuroImage 24:332-338.

Costantini M, Galati G, Ferretti A, Caulo M, Tartaro A, Romani GL, Aglioti SM (2005) Neural systems underlying observation of humanly impossible movements: an FMRI study. Cereb Cortex 15:1761-1767.

Creem-Regehr SH, Lee JN (2005) Neural representations of graspable objects: are tools special? Brain research Cognitive brain research 22:457-469.

Cremers J, Dessoullieres A, Garraux G (2012) Hemispheric specialization during mental imagery of brisk walking. Human brain mapping 33:873-882.

Cutting LE, Clements AM, Courtney S, Rimrodt SL, Schafer JG, Bisesi J, Pekar JJ, Pugh KR (2006) Differential components of sentence comprehension: beyond single word reading and memory. NeuroImage 29:429-438.

D'Arcy RC, Ryner L, Richter W, Service E, Connolly JF (2004) The fan effect in fMRI: left hemisphere specialization in verbal working memory. Neuroreport 15:1851-1855.

David N, Bewernick BH, Cohen MX, Newen A, Lux S, Fink GR, Shah NJ, Vogeley K (2006) Neural representations of self versus other: visual-spatial perspective taking and agency in a virtual ball-tossing game. Journal of cognitive neuroscience 18:898-910.

Deng Y, Booth JR, Chou TL, Ding GS, Peng DL (2008) Item-specific and generalization effects on brain activation when learning Chinese characters. Neuropsychologia 46:1864-1876.

Dieterich M, Muller-Schunk S, Stephan T, Bense S, Seelos K, Yousry TA (2009) Functional magnetic resonance imaging activations of cortical eye fields during saccades, smooth pursuit, and optokinetic nystagmus. Annals of the New York Academy of Sciences 1164:282-292.

Dong Y, Nakamura K, Okada T, Hanakawa T, Fukuyama H, Mazziotta JC, Shibasaki H (2005) Neural mechanisms underlying the processing of Chinese words: an fMRI study. Neuroscience research 52:139-145.

Donnelly KM, Allendorfer JB, Szaflarski JP (2011) Right hemispheric participation in semantic decision improves performance. Brain research 1419:105-116.

Doricchi F, Macci E, Silvetti M, Macaluso E (2010) Neural correlates of the spatial and expectancy components of endogenous and stimulus-driven orienting of attention in the Posner task. Cereb Cortex 20:1574-1585.

Dormal V, Andres M, Dormal G, Pesenti M (2010) Mode-dependent and mode-independent representations of numerosity in the right intraparietal sulcus. NeuroImage 52:1677-1686.

Downing PE, Peelen MV, Wiggett AJ, Tew BD (2006) The role of the extrastriate body area in action perception. Social neuroscience 1:52-62.

Esterman M, Prinzmetal W, DeGutis J, Landau A, Hazeltine E, Verstynen T, Robertson L (2008) Voluntary and involuntary attention affect face discrimination differently. Neuropsychologia 46:1032-1040.

Fan J, McCandliss BD, Fossella J, Flombaum JI, Posner MI (2005) The activation of attentional networks. NeuroImage 26:471-479.

Fehr T, Code C, Herrmann M (2007) Common brain regions underlying different arithmetic operations as revealed by conjunct fMRI-BOLD activation. Brain research 1172:93-102.

Fehr T, Code C, Herrmann M (2008) Auditory task presentation reveals predominantly right hemispheric fMRI activation patterns during mental calculation. Neuroscience letters 431:39-44.

Filippi M, Riccitelli G, Falini A, Di Salle F, Vuilleumier P, Comi G, Rocca MA (2010) The brain functional networks associated to human and animal suffering differ among omnivores, vegetarians and vegans. PloS one 5:e10847.

Fusser F, Linden DE, Rahm B, Hampel H, Haenschel C, Mayer JS (2011) Common capacity-limited neural mechanisms of selective attention and spatial working memory encoding. The European journal of neuroscience 34:827-838.

Garavan H, Hester R, Murphy K, Fassbender C, Kelly C (2006) Individual differences in the functional neuroanatomy of inhibitory control. Brain research 1105:130-142.

Gheysen F, Van Opstal F, Roggeman C, Van Waelvelde H, Fias W (2010) Hippocampal contribution to early and later stages of implicit motor sequence learning. Experimental brain research Experimentelle Hirnforschung Experimentation cerebrale 202:795-807.

Ghosh S, Basu A, Kumaran SS, Khushu S (2010) Functional mapping of language networks in the normal brain using a word-association task. The Indian journal of radiology & imaging 20:182-187.

Gobel SM, Johansen-Berg H, Behrens T, Rushworth MF (2004) Response-selection-related parietal activation during number comparison. Journal of cognitive neuroscience 16:1536-1551.

Gogos A, Gavrilescu M, Davison S, Searle K, Adams J, Rossell SL, Bell R, Davis SR, Egan GF (2010) Greater superior than inferior parietal lobule activation with increasing rotation angle during mental rotation: an fMRI study. Neuropsychologia 48:529-535.

Grabner RH, Ischebeck A, Reishofer G, Koschutnig K, Delazer M, Ebner F, Neuper C (2009) Fact learning in complex arithmetic and figural-spatial tasks: the role of the angular gyrus and its relation to mathematical competence. Human brain mapping 30:2936-2952.

Grezes J, Armony JL, Rowe J, Passingham RE (2003) Activations related to "mirror" and "canonical" neurones in the human brain: an fMRI study. NeuroImage 18:928-937.

Grindrod CM, Bilenko NY, Myers EB, Blumstein SE (2008) The role of the left inferior frontal gyrus in implicit semantic competition and selection: An event-related fMRI study. Brain research 1229:167-178.

Gullick MM, Temple E (2011) Are historic years understood as numbers or events? An fMRI study of numbers with semantic associations. Brain and cognition 77:356-364.

Gur RC, Turetsky BI, Loughead J, Waxman J, Snyder W, Ragland JD, Elliott MA, Bilker WB, Arnold SE, Gur RE (2007) Hemodynamic responses in neural circuitries for detection of visual target and novelty: An event-related fMRI study. Human brain mapping 28:263-274.

Hahn B, Ross TJ, Stein EA (2006) Neuroanatomical dissociation between bottom-up and top-down processes of visuospatial selective attention. NeuroImage 32:842-853.

Halko ML, Hlushchuk Y, Hari R, Schurmann M (2009) Competing with peers: mentalizing-related brain activity reflects what is at stake. NeuroImage 46:542-548.

Hanakawa T, Honda M, Sawamoto N, Okada T, Yonekura Y, Fukuyama H, Shibasaki H (2002) The role of rostral Brodmann area 6 in mental-operation tasks: an integrative neuroimaging approach. Cereb Cortex 12:1157-1170.

Harrington DL, Boyd LA, Mayer AR, Sheltraw DM, Lee RR, Huang M, Rao SM (2004) Neural representation of interval encoding and decision making. Brain research Cognitive brain research 21:193-205.

Harrington GS, Farias D, Davis CH (2009) The neural basis for simulated drawing and the semantic implications. Cortex; a journal devoted to the study of the nervous system and behavior 45:386-393.

Haslinger B, Erhard P, Weilke F, Ceballos-Baumann AO, Bartenstein P, Grafin von Einsiedel H, Schwaiger M, Conrad B, Boecker H (2002) The role of lateral premotor-cerebellar-parietal circuits in motor sequence control: a parametric fMRI study. Brain research Cognitive brain research 13:159-168.

Hennenlotter A, Schroeder U, Erhard P, Castrop F, Haslinger B, Stoecker D, Lange KW, Ceballos-Baumann AO (2005) A common neural basis for receptive and expressive communication of pleasant facial affect. NeuroImage 26:581-591.

Hoenig K, Scheef L (2009) Neural correlates of semantic ambiguity processing during context verification. NeuroImage 45:1009-1019.

Holle H, Gunter TC, Ruschemeyer SA, Hennenlotter A, Iacoboni M (2008) Neural correlates of the processing of co-speech gestures. NeuroImage 39:2010-2024.

Hosseini SM, Rostami M, Yomogida Y, Takahashi M, Tsukiura T, Kawashima R (2010) Aging and decision making under uncertainty: behavioral and neural evidence for the preservation of decision making in the absence of learning in old age. NeuroImage 52:1514-1520.

Hu S, Li CS (2012) Neural processes of preparatory control for stop signal inhibition. Human brain mapping 33:2785-2796.

Hwang K, Palmer ED, Basho S, Zadra JR, Muller RA (2009) Category-specific activations during word generation reflect experiential sensorimotor modalities. NeuroImage 48:717-725.

Iacoboni M, Lieberman MD, Knowlton BJ, Molnar-Szakacs I, Moritz M, Throop CJ, Fiske AP (2004) Watching social interactions produces dorsomedial prefrontal and medial parietal BOLD fMRI signal increases compared to a resting baseline. NeuroImage 21:1167-1173.

Ischebeck A, Zamarian L, Siedentopf C, Koppelstatter F, Benke T, Felber S, Delazer M (2006) How specifically do we learn? Imaging the learning of multiplication and subtraction. NeuroImage 30:1365-1375.

Jarcho JM, Berkman ET, Lieberman MD (2011) The neural basis of rationalization: cognitive dissonance reduction during decision-making. Social cognitive and affective neuroscience 6:460-467.

Jonas M, Siebner HR, Biermann-Ruben K, Kessler K, Baumer T, Buchel C, Schnitzler A, Munchau A (2007) Do simple intransitive finger movements consistently activate frontoparietal mirror neuron areas in humans? NeuroImage 36 Suppl 2:T44-53.

Jost K, Khader P, Burke M, Bien S, Rosler F (2009) Dissociating the solution processes of small, large, and zero multiplications by means of fMRI. NeuroImage 46:308-318.

Jost K, Khader PH, Burke M, Bien S, Rosler F (2011) Frontal and parietal contributions to arithmetic fact retrieval: a parametric analysis of the problem-size effect. Human brain mapping 32:51-59.

Kaas A, Weigelt S, Roebroeck A, Kohler A, Muckli L (2010) Imagery of a moving object: the role of occipital cortex and human MT/V5+. NeuroImage 49:794-804.

Kahnt T, Grueschow M, Speck O, Haynes JD (2011) Perceptual learning and decision-making in human medial frontal cortex. Neuron 70:549-559.

Keightley ML, Chiew KS, Winocur G, Grady CL (2007) Age-related differences in brain activity underlying identification of emotional expressions in faces. Social cognitive and affective neuroscience 2:292-302.

Keightley ML, Chiew KS, Anderson JA, Grady CL (2011) Neural correlates of recognition memory for emotional faces and scenes. Social cognitive and affective neuroscience 6:24-37.

Kemmerer D, Castillo JG, Talavage T, Patterson S, Wiley C (2008) Neuroanatomical distribution of five semantic components of verbs: evidence from fMRI. Brain and language 107:16-43.

Kessler H, Doyen-Waldecker C, Hofer C, Hoffmann H, Traue HC, Abler B (2011) Neural correlates of the perception of dynamic versus static facial expressions of emotion. Psycho-social medicine 8:Doc03.

Kircher T, Sass K, Sachs O, Krach S (2009) Priming words with pictures: neural correlates of semantic associations in a cross-modal priming task using fMRI. Human brain mapping 30:4116-4128.

Kircher T, Nagels A, Kirner-Veselinovic A, Krach S (2011) Neural correlates of rhyming vs. lexical and semantic fluency. Brain research 1391:71-80.

Kirschen MP, Chen SH, Desmond JE (2010) Modality specific cerebro-cerebellar activations in verbal working memory: an fMRI study. Behavioural neurology 23:51-63.

Kirschen MP, Chen SH, Schraedley-Desmond P, Desmond JE (2005) Load- and practice-dependent increases in cerebro-cerebellar activation in verbal working memory: an fMRI study. NeuroImage 24:462-472.

Kitada R, Johnsrude IS, Kochiyama T, Lederman SJ (2010) Brain networks involved in haptic and visual identification of facial expressions of emotion: an fMRI study. NeuroImage 49:1677-1689.

Kong J, Wang C, Kwong K, Vangel M, Chua E, Gollub R (2005) The neural substrate of arithmetic operations and procedure complexity. Brain research Cognitive brain research 22:397-405.

Koyama M, Hasegawa I, Osada T, Adachi Y, Nakahara K, Miyashita Y (2004) Functional magnetic resonance imaging of macaque monkeys performing visually guided saccade tasks: comparison of cortical eye fields with humans. Neuron 41:795-807.

Krach S, Hegel F, Wrede B, Sagerer G, Binkofski F, Kircher T (2008) Can machines think? Interaction and perspective taking with robots investigated via fMRI. PloS one 3:e2597.

Kuhn S, Bodammer NC, Brass M (2010) Dissociating mental states related to doing nothing by means of fMRI pattern classification. NeuroImage 53:1294-1300.

la Fougere C, Zwergal A, Rominger A, Forster S, Fesl G, Dieterich M, Brandt T, Strupp M, Bartenstein P, Jahn K (2010) Real versus imagined locomotion: a [18F]-FDG PET-fMRI comparison. NeuroImage 50:1589-1598.

Lee KM, Wade AR, Lee BT (2006) Differential correlation of frontal and parietal activity with the number of alternatives for cued choice saccades. NeuroImage 33:307-315.

Leung HC, Oh H, Ferri J, Yi Y (2007) Load response functions in the human spatial working memory circuit during location memory updating. NeuroImage 35:368-377.

Linden DE, Bittner RA, Muckli L, Waltz JA, Kriegeskorte N, Goebel R, Singer W, Munk MH (2003) Cortical capacity constraints for visual working memory: dissociation of fMRI load effects in a fronto-parietal network. NeuroImage 20:1518-1530.

Logie RH, Venneri A, Della Sala S, Redpath TW, Marshall I (2003) Brain activation and the phonological loop: the impact of rehearsal. Brain and cognition 53:293-296.

Lui F, Buccino G, Duzzi D, Benuzzi F, Crisi G, Baraldi P, Nichelli P, Porro CA, Rizzolatti G (2008) Neural substrates for observing and imagining non-object-directed actions. Social neuroscience 3:261-275.

Lycke C, Specht K, Ersland L, Hugdahl K (2008) An fMRI study of phonological and spatial working memory using identical stimuli. Scandinavian journal of psychology 49:393-301.

Macaluso E, Frith CD, Driver J (2007) Delay activity and sensory-motor translation during planned eye or hand movements to visual or tactile targets. Journal of neurophysiology 98:3081-3094.

Mao L, Zhou B, Zhou W, Han S (2007) Neural correlates of covert orienting of visual spatial attention along vertical and horizontal dimensions. Brain research 1136:142-153.

Marvel CL, Desmond JE (2012) From storage to manipulation: How the neural correlates of verbal working memory reflect varying demands on inner speech. Brain and language 120:42-51.

Mason RA, Just MA (2011) Differentiable cortical networks for inferences concerning people's intentions versus physical causality. Human brain mapping 32:313-329.

Matsuda T, Matsuura M, Ohkubo T, Ohkubo H, Matsushima E, Inoue K, Taira M, Kojima T (2004) Functional MRI mapping of brain activation during visually guided saccades and antisaccades: cortical and subcortical networks. Psychiatry research 131:147-155.

McMillan CT, Clark R, Moore P, Devita C, Grossman M (2005) Neural basis for generalized quantifier comprehension. Neuropsychologia 43:1729-1737.

McMillan CT, Clark R, Gunawardena D, Ryant N, Grossman M (2012) fMRI evidence for strategic decision-making during resolution of pronoun reference. Neuropsychologia 50:674-687.

McNab F, Leroux G, Strand F, Thorell L, Bergman S, Klingberg T (2008) Common and unique components of inhibition and working memory: an fMRI, within-subjects investigation. Neuropsychologia 46:2668-2682.

Meister IG, Iacoboni M (2007) No language-specific activation during linguistic processing of observed actions. PloS one 2:e891.

Mizuhara H, Yamaguchi Y (2007) Human cortical circuits for central executive function emerge by theta phase synchronization. NeuroImage 36:232-244.

Morgan HM, Jackson MC, Klein C, Mohr H, Shapiro KL, Linden DE (2010) Neural signatures of stimulus features in visual working memory--a spatiotemporal approach. Cereb Cortex 20:187-197.

Muller K, Kleiser R, Mechsner F, Seitz RJ (2011a) Involvement of area MT in bimanual finger movements in left-handers: an fMRI study. The European journal of neuroscience 34:1301-1309.

Muller VI, Habel U, Derntl B, Schneider F, Zilles K, Turetsky BI, Eickhoff SB (2011b) Incongruence effects in crossmodal emotional integration. NeuroImage 54:2257-2266.

Munoz-Torres Z, Armony JL, Trejo-Martinez D, Conde R, Corsi-Cabrera M (2011) Behavioural and neural effects of diazepam on a rule-guided response selection task. Neuroscience research 70:260-268.

Nakai T, Matsuo K, Ohgami Y, Oishi K, Kato C (2005) An fMRI study of temporal sequencing of motor regulation guided by an auditory cue--a comparison with visual guidance. Cognitive processing 6:128-135.

Natale E, Marzi CA, Girelli M, Pavone EF, Pollmann S (2006) ERP and fMRI correlates of endogenous and exogenous focusing of visual-spatial attention. The European journal of neuroscience 23:2511-2521.

Nelles G, de Greiff A, Pscherer A, Esser J (2009) Age-related differences of saccade induced cortical activation. Neuroscience letters 458:15-18.

Newman SD, Lee D, Christopher Bates L (2007) The timecourse of activation within the cortical network associated with visual imagery. The open neuroimaging journal 1:1-9.

O'Boyle MW, Cunnington R, Silk TJ, Vaughan D, Jackson G, Syngeniotis A, Egan GF (2005) Mathematically gifted male adolescents activate a unique brain network during mental rotation. Brain research Cognitive brain research 25:583-587.

O'Hare ED, Lu LH, Houston SM, Bookheimer SY, Sowell ER (2008) Neurodevelopmental changes in verbal working memory load-dependency: an fMRI investigation. NeuroImage 42:1678-1685.

Ortigue S, Thompson JC, Parasuraman R, Grafton ST (2009) Spatio-temporal dynamics of human intention understanding in temporo-parietal cortex: a combined EEG/fMRI repetition suppression paradigm. PloS one 4:e6962.

Park JY, Gu BM, Kang DH, Shin YW, Choi CH, Lee JM, Kwon JS (2010) Integration of cross-modal emotional information in the human brain: an fMRI study. Cortex; a journal devoted to the study of the nervous system and behavior 46:161-169.

Peelle JE, Troiani V, Grossman M (2009) Interaction between process and content in semantic memory: an fMRI study of noun feature knowledge. Neuropsychologia 47:995-1003.

Pierno AC, Becchio C, Tubaldi F, Turella L, Castiello U (2008) Motor ontology in representing gaze-object relations. Neuroscience letters 430:246-251.

Rabin JS, Gilboa A, Stuss DT, Mar RA, Rosenbaum RS (2010) Common and unique neural correlates of autobiographical memory and theory of mind. Journal of cognitive neuroscience 22:1095-1111.

Rektor I, Sochurkova D, Bockova M (2006) Intracerebral ERD/ERS in voluntary movement and in cognitive visuomotor task. Progress in brain research 159:311-330.

Remy F, Wenderoth N, Lipkens K, Swinnen SP (2010) Dual-task interference during initial learning of a new motor task results from competition for the same brain areas. Neuropsychologia 48:2517-2527.

Reuter B, Kaufmann C, Bender J, Pinkpank T, Kathmann N (2010) Distinct neural correlates for volitional generation and inhibition of saccades. Journal of cognitive neuroscience 22:728-738.

Ruff CC, Driver J (2006) Attentional preparation for a lateralized visual distractor: behavioral and fMRI evidence. Journal of cognitive neuroscience 18:522-538.

Ruff S, Cardebat D, Marie N, Demonet JF (2002) Enhanced response of the left frontal cortex to slowed down speech in dyslexia: an fMRI study. Neuroreport 13:1285-1289.

Ruge H, Muller SC, Braver TS (2010) Anticipating the consequences of action: an fMRI study of intention-based task preparation. Psychophysiology 47:1019-1027.

Samson AC, Zysset S, Huber O (2008) Cognitive humor processing: different logical mechanisms in nonverbal cartoons--an fMRI study. Social neuroscience 3:125-140.

Sass K, Habel U, Sachs O, Huber W, Gauggel S, Kircher T (2012) The influence of emotional associations on the neural correlates of semantic priming. Human brain mapping 33:676-694.

Schafer RJ, Page KA, Arora J, Sherwin R, Constable RT (2012) BOLD response to semantic and syntactic processing during hypoglycemia is load-dependent. Brain and language 120:1-14.

Scheibe C, Ullsperger M, Sommer W, Heekeren HR (2010) Effects of parametrical and trial-to-trial variation in prior probability processing revealed by simultaneous electroencephalogram/functional magnetic resonance imaging. The Journal of neuroscience : the official journal of the Society for Neuroscience 30:16709-16717.

Scheuerecker J, Frodl T, Koutsouleris N, Zetzsche T, Wiesmann M, Kleemann AM, Bruckmann H, Schmitt G, Moller HJ, Meisenzahl EM (2007) Cerebral differences in explicit and implicit emotional processing--an fMRI study. Neuropsychobiology 56:32-39.

Schneiders JA, Opitz B, Krick CM, Mecklinger A (2011) Separating intra-modal and across-modal training effects in visual working memory: an fMRI investigation. Cereb Cortex 21:2555-2564.

Schulz KP, Clerkin SM, Halperin JM, Newcorn JH, Tang CY, Fan J (2009) Dissociable neural effects of stimulus valence and preceding context during the inhibition of responses to emotional faces. Human brain mapping 30:2821-2833.

Seghier ML, Lazeyras F, Pegna AJ, Annoni JM, Zimine I, Mayer E, Michel CM, Khateb A (2004) Variability of fMRI activation during a phonological and semantic language task in healthy subjects. Human brain mapping 23:140-155.

Serences JT (2008) Value-based modulations in human visual cortex. Neuron 60:1169-1181.

Seurinck R, Vingerhoets G, Vandemaele P, Deblaere K, Achten E (2005) Trial pacing in mental rotation tasks. NeuroImage 25:1187-1196.

Simon SR, Meunier M, Piettre L, Berardi AM, Segebarth CM, Boussaoud D (2002) Spatial attention and memory versus motor preparation: premotor cortex involvement as revealed by fMRI. Journal of neurophysiology 88:2047-2057.

Sommer M, Rothmayr C, Dohnel K, Meinhardt J, Schwerdtner J, Sodian B, Hajak G (2010) How should I decide? The neural correlates of everyday moral reasoning. Neuropsychologia 48:2018-2026.

Strand F, Forssberg H, Klingberg T, Norrelgen F (2008) Phonological working memory with auditory presentation of pseudo-words -- an event related fMRI Study. Brain research 1212:48-54.

Straube B, Green A, Jansen A, Chatterjee A, Kircher T (2010) Social cues, mentalizing and the neural processing of speech accompanied by gestures. Neuropsychologia 48:382-393.

Talsma D, Coe B, Munoz DP, Theeuwes J (2010) Brain structures involved in visual search in the presence and absence of color singletons. Journal of cognitive neuroscience 22:761-774.

Tham WW, Rickard Liow SJ, Rajapakse JC, Choong Leong T, Ng SE, Lim WE, Ho LG (2005) Phonological processing in Chinese-English bilingual biscriptals: an fMRI study. NeuroImage 28:579-587.

Thuy DH, Matsuo K, Nakamura K, Toma K, Oga T, Nakai T, Shibasaki H, Fukuyama H (2004) Implicit and explicit processing of kanji and kana words and non-words studied with fMRI. NeuroImage 23:878-889.

Toepper M, Markowitsch HJ, Gebhardt H, Beblo T, Thomas C, Gallhofer B, Driessen M, Sammer G (2010) Hippocampal involvement in working memory encoding of changing locations: an fMRI study. Brain research 1354:91-99.

Vallesi A, McIntosh AR, Crescentini C, Stuss DT (2012) fMRI investigation of speed-accuracy strategy switching. Human brain mapping 33:1677-1688.

van der Meer L, Groenewold NA, Nolen WA, Pijnenborg M, Aleman A (2011) Inhibit yourself and understand the other: neural basis of distinct processes underlying Theory of Mind. NeuroImage 56:2364-2374.

Wager TD, Sylvester CY, Lacey SC, Nee DE, Franklin M, Jonides J (2005) Common and unique components of response inhibition revealed by fMRI. NeuroImage 27:323-340.

Walter E, Dassonville P (2011) Activation in a frontoparietal cortical network underlies individual differences in the performance of an embedded figures task. PloS one 6:e20742.

Wang L, Huettel S, De Bellis MD (2008) Neural substrates for processing task-irrelevant sad images in adolescents. Developmental science 11:23-32.

Watanabe J, Sugiura M, Sato K, Sato Y, Maeda Y, Matsue Y, Fukuda H, Kawashima R (2002) The human prefrontal and parietal association cortices are involved in NO-GO performances: an event-related fMRI study. NeuroImage 17:1207-1216.

Weidner R, Krummenacher J, Reimann B, Muller HJ, Fink GR (2009) Sources of top-down control in visual search. Journal of cognitive neuroscience 21:2100-2113.

Weissman DH, Woldorff MG, Hazlett CJ, Mangun GR (2002) Effects of practice on executive control investigated with fMRI. Brain research Cognitive brain research 15:47-60.

Welcome SE, Joanisse MF (2012) Individual differences in skilled adult readers reveal dissociable patterns of neural activity associated with component processes of reading. Brain and language 120:360-371.

Wendelken C, Ditterich J, Bunge SA, Carter CS (2009) Stimulus and response conflict processing during perceptual decision making. Cognitive, affective & behavioral neuroscience 9:434-447.

Wildgruber D, Riecker A, Hertrich I, Erb M, Grodd W, Ethofer T, Ackermann H (2005) Identification of emotional intonation evaluated by fMRI. NeuroImage 24:1233-1241.

Woolgar A, Thompson R, Bor D, Duncan J (2011) Multi-voxel coding of stimuli, rules, and responses in human frontoparietal cortex. NeuroImage 56:744-752.

Yi-Rong N, Si-Yun S, Zhou-Yi G, Si-Run L, Yun B, Song-Hao L, Chan WY (2011) Dissociated brain organization for two-digit addition and subtraction: an fMRI investigation. Brain research bulletin 86:395-402.

Yokoyama S, Watanabe J, Iwata K, Ikuta N, Haji T, Usui N, Taira M, Miyamoto T, Nakamura W, Sato S, Horie K, Kawashima R (2007) Is Broca's area involved in the processing of passive sentences? An event-related fMRI study. Neuropsychologia 45:989-996.

Yokoyama S, Okamoto H, Miyamoto T, Yoshimoto K, Kim J, Iwata K, Jeong H, Uchida S, Ikuta N, Sassa Y, Nakamura W, Horie K, Sato S, Kawashima R (2006) Cortical activation in the processing of passive sentences in L1 and L2: an fMRI study. NeuroImage 30:570-579.

Zhao J, Li QL, Wang JJ, Yang Y, Deng Y, Bi HY (2012) Neural basis of phonological processing in second language reading: an fMRI study of Chinese regularity effect. NeuroImage 60:419-425.
